# Lipid production by the oleaginous yeast *Yarrowia lipolytica* using industrial by-products under different culture conditions

**DOI:** 10.1186/s13068-015-0286-z

**Published:** 2015-07-25

**Authors:** Magdalena Rakicka, Zbigniew Lazar, Thierry Dulermo, Patrick Fickers, Jean Marc Nicaud

**Affiliations:** INRA, UMR1319 Micalis, 78350, Jouy-en-Josas, France; AgroParisTech, UMR Micalis, Jouy-en-Josas, France; Department of Biotechnology and Food Microbiology, Wrocław University of Environmental and Life Sciences, Chełmońskiego Str. 37/41, 51-630 Wrocław, Poland; Microbial Processes and Interactions, Gembloux Agro Bio-Tech, Université de Liège, Passage des Déportés, 2, 5030 Gembloux, Belgium; Institut Micalis, INRA-AgroParisTech, UMR1319, Team BIMLip: Biologie Intégrative du Métabolisme Lipidique, CBAI, 78850 Thiverval-Grignon, France

**Keywords:** *Yarrowia lipolytica*, Oleaginous yeast, Biolipid production, Crude glycerol, Molasses, Continuous culture, Triglyceride

## Abstract

**Background:**

Microbial lipid production using renewable feedstock shows great promise for the biodiesel industry.

**Results:**

In this study, the ability of a lipid-engineered *Yarrowia lipolytica* strain JMY4086 to produce lipids using molasses and crude glycerol under different oxygenation conditions and at different inoculum densities was evaluated in fed-batch cultures. The greatest lipid content, 31% of CDW, was obtained using a low-density inoculum, a constant agitation rate of 800 rpm, and an oxygenation rate of 1.5 L/min. When the strain was cultured for 450 h in a chemostat containing a nitrogen-limited medium (dilution rate of 0.01 h^−1^; 250 g/L crude glycerol), volumetric lipid productivity was 0.43 g/L/h and biomass yield was 60 g CDW/L. The coefficient of lipid yield to glycerol consumption (*Y*_*L*/gly_) and the coefficient of lipid yield to biomass yield (*Y*_*L*/*X*_) were equal to 0.1 and 0.4, respectively.

**Conclusions:**

These results indicate that lipids may be produced using renewable feedstock, thus providing a means of decreasing the cost of biodiesel production. Furthermore, using molasses for biomass production and recycling glycerol from the biodiesel industry should allow biolipids to be sustainably produced.

## Background

The distinct possibility of fossil fuel depletion is currently forcing the fuel industry to develop alternative energy sources, such as biodiesel [1]. Because biodiesel is derived from vegetable oils, there is competition between biodiesel producers and food crop farmers for arable lands [2]. Consequently, one of the industry’s goals is to find novel ways of producing biodiesel. One possible strategy involves the transformation of waste materials and/or co-products, such as whey, crop residues, crude glycerol, or crude fats, into triglycerides or fatty acids using microbial cell factories [3, 4]. These processes are advantageous compared to conventional methods, since they use waste materials generated by various industries as feedstock. Moreover, microbial lipid can be produced in close proximity to biodiesel industrial plants and it is easy to scale up their production [5].

Different bacteria, yeasts, algae, and fungi have the ability to convert carbohydrates and other substrates into intracellular lipid. When a microorganism’s intracellular lipid accumulation levels are greater than 20% of cell dry weight (CDW), it is labeled an “oleaginous microorganism”. Oleaginous microorganisms include yeast species, such as *Rhodosporidium* sp., *Rhodotorula* sp., *Lipomyces* sp., and *Yarrowia lipolytica*, whose intracellular lipid accumulation levels can reach 80% of CDW [6–8]. The main components of the accumulated lipid are triacylglycerols composed of long-chain fatty acids (16–18 carbon atoms in the chain) [6–8].

There are many ways of increasing intracellular lipid accumulation. Some involve metabolically engineering microbial strains to either improve their lipid storage capacities or synthesize lipids with specific fatty acid profiles [8–11]. Others focus on refining the production process by identifying optimal culture conditions and defining optimal medium composition [12–14]. For instance, fed-batch culturing is the most convenient system in pilot experiments seeking to establish optimal production conditions: it helps identify the best medium composition and any supplements needed. However, continuous cultures are also of great interest when the goal is to enhance lipid accumulation levels, especially those of yeast grown as well-dispersed, non-filamentous cells [15].

Due to *Y. lipolytica*’s unique physiological characteristics (i.e., its ability to metabolize hydrophobic substrates such as alkanes, fatty acids, and lipids), its ability to accumulate high levels of lipids, and its suite of efficient genetic tools [16], this yeast is a model organism for biolipid production and it is thought to have great applied potential [6–8, 11], both in the production of typical biofuel lipids [9–11] and oils with unusual fatty acid profiles or polyunsaturated fatty acids [3, 4, 17]. In this study, *Y. lipolytica* JMY4086, a strain with an improved lipid accumulation capacity, was used to exploit unpurified, low-cost industrial by-products, such as sugar beet molasses and the crude glycerol produced by the biodiesel industry and lipid production under different culture conditions was quantified. Molasses was used as a source of carbon, minerals, and vitamins, which are crucial for fermentation [18]. Moreover, molasses is used as the main substrate in the production of baker’s yeast, organic acids, amino acids, and acetone/butanol [15]. In yeast, the glycolytic pathway produces intermediate compounds from glycerol either via the phosphorylation pathway [19, 20] or the oxidative pathway (dehydrogenation of glycerol and the subsequent phosphorylation of the reaction product) [21]. Dihydroxyacetone phosphate, the product of these reactions, can subsequently be converted into citric acid, storage lipids, or various other products [22, 23]. Additionally, glycerol may be readily incorporated in the core of triglycerides, which are stored in lipid bodies along with steryl esters [10].

The aim of this study was to produce valuable information that could be used in future research examining the biotransformation of crude glycerol into triglycerides (TAGs) with a view to producing biolipids, also known as single-cell oils (SCOs). This process may serve as an alternative means of decreasing biodiesel production costs while simultaneously recycling glycerol.

## Results and discussion

Previous work has found that *Y. lipolytica* JMY4086 can produce biolipids from substrates such as pure glucose, fructose, and sucrose in batch bioreactors [19]. The present study investigated whether low-cost raw materials such as molasses and crude glycerol could also serve as substrates for biolipid production and accumulation; the substrate concentration, oxygenation conditions, and inoculum densities were varied. Compared to other oleaginous microorganisms, *Y. lipolytica* has the unique ability to accumulate lipids when nitrogen is limited and to remobilize them when carbon is limited [24]. Therefore, all culturing was performed under low nitrogen conditions. Furthermore, TAG remobilization was avoided because the *TGL4* gene, which encodes triglyceride lipase *Yl*Tgl4, was deleted from JMY4086 [17].

### Fed-batch cultures subject to different oxygenation conditions and initiated with different inoculum densities

Studies examining lipid production by *Y. lipolytica* using fed-batch or repeated-batch cultures are scarce. Moreover, only glycerol has been used as a substrate for cell growth and lipid synthesis [25]. This study utilized a two-step process: biomass was produced using molasses for 48 h, and then lipids were produced using glycerol as the main carbon source. Biomass yield and lipid production were analyzed at two different inoculum densities (low density and high density) and under two sets of oxygenation conditions (unregulated and regulated). In the unregulated strategy “Oxy-const”, dissolved oxygen (DO) was not regulated; in the regulated strategy “Oxy-regul”, DO was regulated at 50% saturation (see “Methods”).

**Figure 1:**
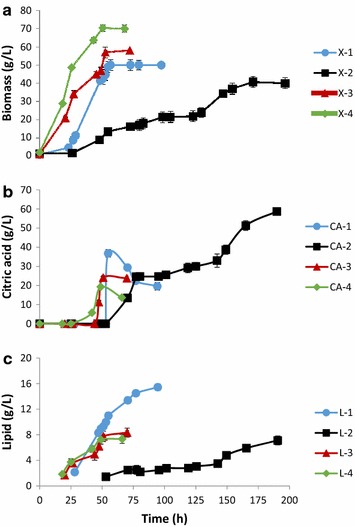
Effect of oxygenation conditions and inoculation densities on growth, citric acid production, and lipid production by *Y. lipolytica* grown in molasses. Strain JMY4086 was grown in a molasses medium and fed with crude glycerol. Growth is expressed as **a** cell dry weight, **b** citric acid production, and **c** lipid production. *X* biomass, *CA* citric acid, *L* lipids, *1* low-density inoculum/unregulated oxygenation condition (*filled circles*), *2* low-density inoculum/regulated oxygenation condition (*filled squares*), *3* high-density inoculum/unregulated oxygenation condition (*filled triangles*), *4* high-density inoculum/regulated oxygenation condition (*filled diagonals*). The low-density and high-density inocula had optical densities of OD_600_ = 1 and OD_600_ = 6, respectively. For the unregulated oxygenation condition, stirring speed was a constant 800 rpm and the aeration rate was 1.5 L/min. For the regulated oxygenation condition, dissolved oxygen was maintained at 50% saturation and the aeration rate was 0–3.5 L/min. All the results presented are the mean values ± SD for two independent biological replicates.

**Figure 2:**
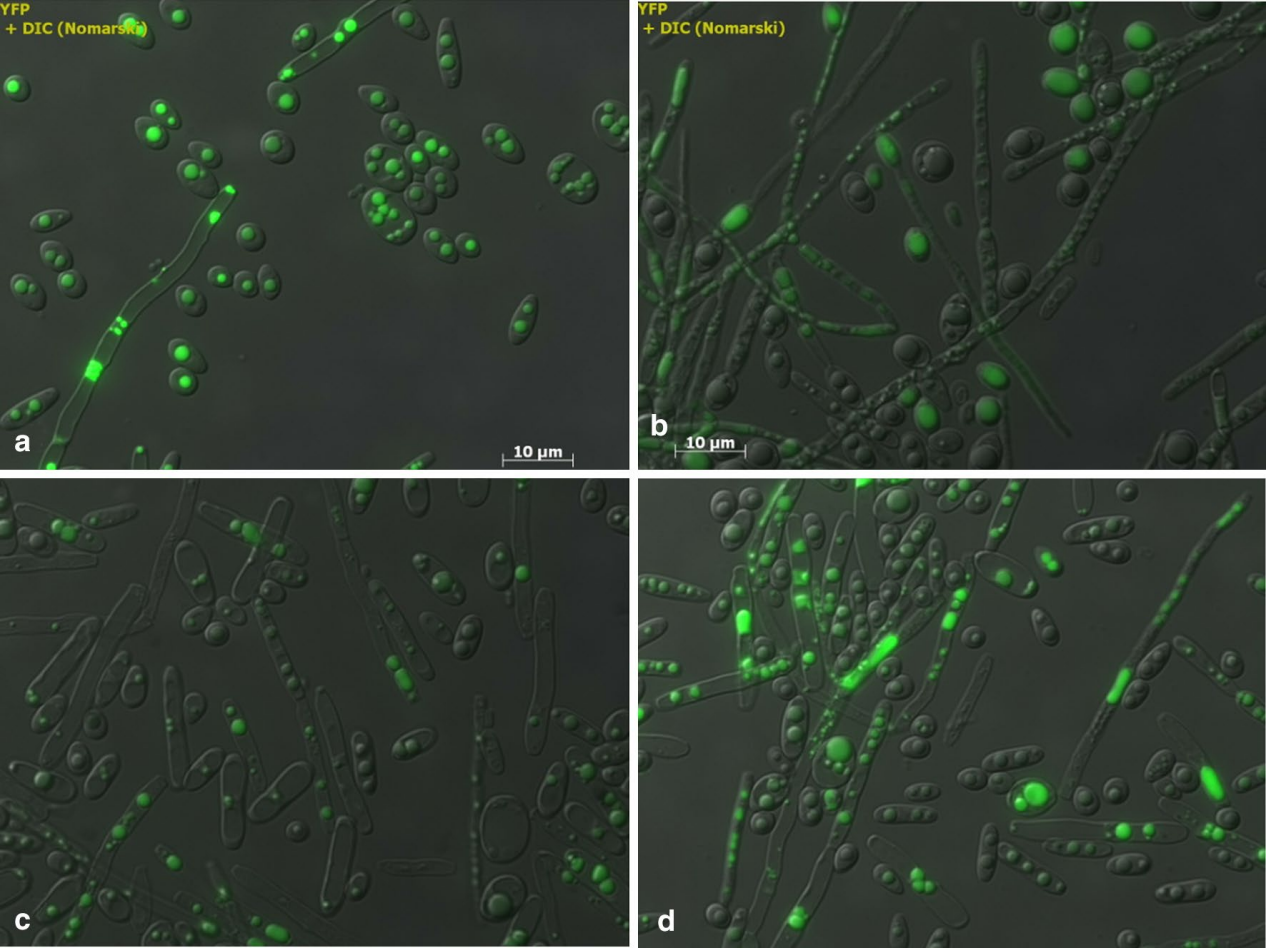
Visualization of JMY4086 cell morphology and lipid bodies at the end of the fed-batch culturing experiment. Images are of cultures from the **a** low-density inoculum/unregulated oxygenation condition, **b** low-density inoculum/regulated oxygenation condition, **c** high-density inoculum/unregulated oxygenation condition; and **d** high-density inoculum/regulated oxygenation condition. The lipid bodies were stained with Bodipy^®^.

When Oxy-const strategy and low-density inoculum were used, the biomass reached 50 g CDW/L and citric acid production was 36.8 g/L after 55 h of culture (Figure 1a, b). During the glycerol-feeding phase, cells converted the citric acid produced into lipids. In those conditions, total lipid concentration increased from 11 to 15.5 g/L (Figure 1c). Yeast lipid content reached 31% of CDW, which corresponds to a volumetric lipid productivity (*Q*_*L*_) of 0.18 g/L/h and a coefficient of lipid yield to glycerol consumption (*Y*_*L*/gly_) of 0.083 g/g (Table 1). This condition also produced a small amount of mycelial cells (Figure 2a). Indeed, low DO levels have been shown to induce the yeast-to-mycelium transition in *Y. lipolytica*. Bellou and colleagues demonstrated that mycelial and/or pseudomycelial forms predominated over the yeast form when DO was low, regardless of the carbon and nitrogen sources used [26].

**Table 1:** Lipid production by *Y. lipolytica* JMY4086 during the glycerol-feeding phase for different oxygenation conditions and inoculation densities

Oxygenation (% dissolved oxygen)	Inoculum density	*Q*_*L*_ (g/L/h)	*Y*_*L*/gly_ (g/g)	*Y*_*L*/*X*_ (g/g)
Oxy-const	Low	0.18	0.083	0.31
Oxy-regul	Low	0.04	0.056	0.17
Oxy-const	High	0.04	0.007	0.14
Oxy-regul	High	0.01	0.002	0.11
Oxy-high	Low	0.17	0.077	0.13

Standard deviations were less than 10% of the mean values.

*Q*_*L*_ volumetric lipid productivity from crude glycerol, *Y*_*L/gly*_ coefficient of lipid yield to glycerol consumption, *Y*_*L/X*_ coefficient of lipid yield to biomass yield.

When oxygenation was regulated and a low-density inoculum was used, cell growth was surprisingly very slow (*r*_*x*_ = 0.24 gCDW/h) and the culture duration (the time required for complete glycerol consumption) was 190 h. Consequently, when crude glycerol was fed into the bioreactor at 48 h, the fructose concentration was still high (30 g/L). In this condition, the biomass yield was lower (40 g/L) because citric acid production was greater (50 g/L) (Figure 1a, b); there was an apparent trade-off between the two processes. The citric acid produced was never reconsumed. The total lipid content was very low, 7 g/L, which corresponds to a *Q*_*L*_ of 0.04 g/L/h (Figure 1c; Table 1). However, *Y*_*L*/gly_ and *Y*_*L*/*X*_ were equal to 0.056 and 0.17 g/g, respectively (Table 1). In these conditions, JMY4086 formed short true mycelia and pseudomycelia (Figure 2b).

High-density inocula were also used under both regulated and unregulated oxygenation conditions. As shown in Figure 1, the lag phase became shorter and culture duration decreased significantly; the latter was 70 and 66 h under regulated and unregulated conditions, respectively. When oxygenation was unregulated, the biomass yield was 58 g/L; it reached 70 g/L when oxygenation was regulated (Figure 1a). Citric acid production was similar across the two conditions (19 and 23 g/L, respectively); however, it was only reconsumed when DO was regulated (Figure 1b). In both cases, compared to the unregulated/low-density condition, *Q*_L_ was low, as were *Y*_*L*/gly_ and *Y*_*L*/*X*_ (Table 1). Furthermore, in both conditions, JMY4086 formed short true mycelia and pseudomycelia (Figure 2c, d).

**Figure 3:**
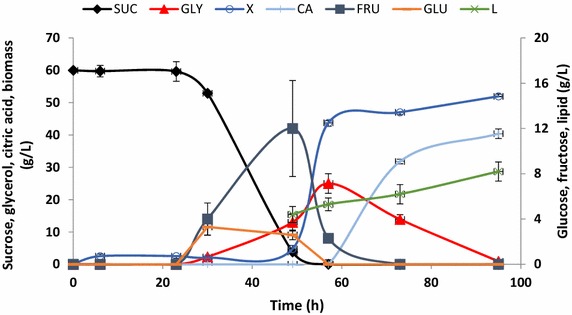
Time course of carbon sources concentration, biomass yield, and lipid and citric acid production during culture of *Y. lipolytica* JMY4086 in the low-density inoculum/high-oxygen experimental conditions. Sucrose (SUC), glucose (GLU), fructose (FRU), glycerol (GLY), biomass yield (*X*), lipid (L), and citric acid (CA). For the high-oxygen condition, the stirring speed was a constant 800 rpm, and the agitation rate was maintained at 3.5 L/min.

Because the fed-batch culture initiated with a low-density inoculum and subject to unregulated oxygenation had the highest lipid production, these conditions were used in a second experiment, in which a higher airflow rate of 3.5 L/min (the high-oxygen condition, “Oxy-high”) was utilized. As a consequence, the lag phase lengthened, sucrose hydrolysis began later—after 30 h (Figure 3)—and lipid accumulation was limited. Citric acid production exceeded 40 g/L, and the compound was not reconsumed (Figure 3). The biomass yield was 59 g/L, and final lipid content was 7.7 g/L, which corresponds to a *Y*_*L*/gly_ of 0.077 g/g (Table 1). These results indicate that increasing the oxygenation rate did not improve yeast growth and lipid production.

The fed-batch experiments revealed that the highest *Q*_*L*_, *Y*_*L*/gly_, and *Y*_*L*/*X*_ values were obtained using a low-density inoculum and unregulated oxygenation. Consequently, these conditions were used in the continuous culture pilot experiment.

### Continuous culture experiment: effects of increasing concentrations of glycerol in the feeding medium

Little research has looked at the synthesis of biolipids from sugars or renewable feedstock by nitrogen-limited continuous cultures [27–29]. Papanikolaou and Aggelis conducted the only study to date to examine biolipid synthesis by *Y. lipolytica* under continuous culture conditions using glycerol as the sole substrate [15].

**Figure 4:**
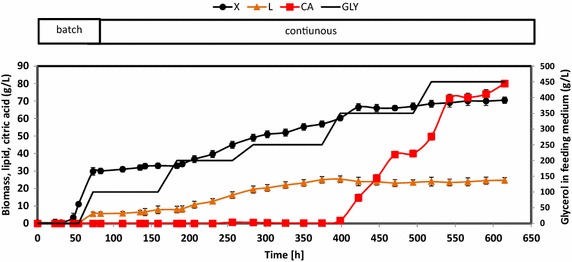
Biomass (*X*), lipid (L), and citric acid (CA) production during SCFB culture of *Y. lipolytica* JMY4086. All the results presented are the mean values ± SD for two independent replicates. The *black line* (GLY) *without symbol* represents the glycerol concentration in the feeding solution.

In this experiment, we used a stepwise continuous fed-batch (SCFB) approach to test the effect of glycerol concentration on lipid production. The cultures started as batch cultures that were grown with molasses to produce biomass; once the sugar supply was exhausted, continuous culturing was initiated. Glycerol was used as feed, and the dilution rate was 0.01 h^−1^. The glycerol concentration in the feeding medium was increased from 100 to 450 g/L in steps that took place every 100 h (Figure 4). It has been shown that the dilution rate and culture-medium C:N ratio strongly affect lipid accumulation [28, 30]. In general, dilution rates of less than 0.06 h^−1^ have been shown to maximize lipid production in continuous cultures across different yeasts [31]. However, a higher dilution rate, of about 0.01 h^−1^, optimizes *Y. lipolytica*’s production of citric acid from glycerol [32]. Therefore, in this experiment, a dilution rate of 0.01 h^−1^ was used. In addition, for JMY4086, lipid accumulation levels were similar across a range of C:N ratios, from 60:1 to 120:1 [19]. Consequently, to maximize cell growth and prevent nitrogen starvation, SCFB culturing was performed using a C:N ratio of 60:1.

Biomass yield and lipid production depended on the glycerol concentration in the feed solution (Figure 4). For glycerol concentrations of 100 g/L, the biomass yield was 32.2 g CDW/L; it reached 67.4 g CDW/L at the highest glycerol concentrations (450 g/L; Table 2). Under such conditions, DO was not limiting, except after 600 h of culture (data not shown). Glycerol was never detected in the culture broth, except at higher feeding concentrations (450 g/L), where glycerol accumulated in the culture broth at a concentration of 0.5 g/L. By comparison, Meesters et al. [33] observed that, in *Cryptococcus curvatus*, cell growth was restricted during lipid accumulation when glycerol concentrations were higher than 64 g/L.

**Table 2:** Characteristics of lipid production in continuous cultures of *Y.* *lipolytica* JMY4086 grown in crude glycerol; SCFB and chemostat culturing were used

Culture method	Glycerol feeding (g/L)	Biomass (gCDW/L)	Residual glycerol (g/L)	Lipid (g/L)	Citric acid (g/L)	*Q*_*L*_ (g/L/h)	*Y*_*L*/gly_ (g/g)	*Y*_*L*/*X*_ (g/g)
SCFB	100	32.2 ± 1.2	nd	6.5 ± 1.1	nd	0.09 ± 0.01	0.13	0.2
SCFB	200	39.1 ± 6.5	nd	14.8 ± 3.9	0.26 ± 0.4	0.26 ± 0.01	0.09	0.37
SCFB	250	49.1 ± 9.6	nd	22.6 ± 2.1	0.26 ± 0.1	0.31 ± 0.01	0.08	0.46
SCFB	350	64.7 ± 2.9	nd	24.1 ± 0.9	24.3 ± 1.6	0.26 ± 0.01	0.06	0.36
SCFB	450	67.4 ± 1.4	0.50 ± 0.05	23.7 ± 1.0	60 ± 1.1	0.25 ± 0.01	0.05	0.35
Chemostat	250	59.8 ± 0.1	nd	24.2 ± 0.1	50.2 ± 0.2	0.43 ± 0.01	0.10	0.4

Mean values for biomass yield, volumetric lipid productivity (*Q*_*L*_), the coefficient of lipid yield to glycerol consumption (*Y*_*L*/gly_), and the coefficient of lipid yield to biomass yield (*Y*_*L*/*X*_) were calculated for the full duration of the different feeding conditions (i.e., different glycerol concentrations).

*nd* not detected.

The highest *Y*_*L*/gly_ value was obtained at a glycerol concentration of 100 g/L. However, since biomass yield was lowest at that concentration, *Q*_*L*_ was also low (0.09 g/L/h). In contrast, when the glycerol concentration was 250 g/L, *Q*_*L*_ and *Y*_*L*/*X*_ were 0.31 g/L/h and 0.46, respectively. At higher glycerol concentrations (350 and 450 g/L), both *Y*_*L*/gly_ and *Y*_*L*/*X*_ were lower (Table 2). During SCFB culturing, very low concentrations of citric acid were present until 400 h. Then, as the glycerol concentration increased to 350 g/L, citric acid started to accumulate; it reached a concentration of 40 g/L (Table 2). This accumulation of citric acid may have resulted from nitrogen limitations or a transition in cell morphology. Indeed, cells occurred in yeast form up until 400 h, at which point they started to filament, forming true mycelia and pseudomycelia (data not shown).

### Lipid production from glycerol in a chemostat culture

**Figure 5:**
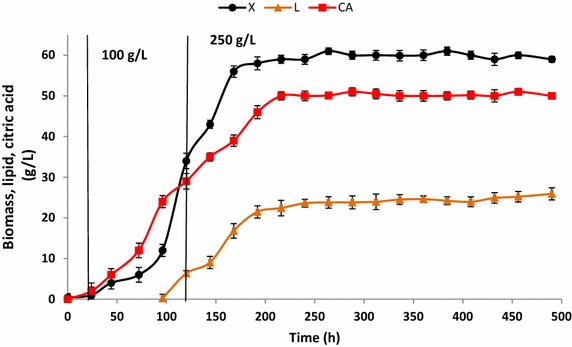
Biomass (*X*), lipid (L), and citric acid (CA) production during the chemostat culture of JMY4086 when 250 g/L of crude glycerol was present in the feeding medium. All the results presented are the mean values ± SD for two independent replicates.

**Figure 6:**
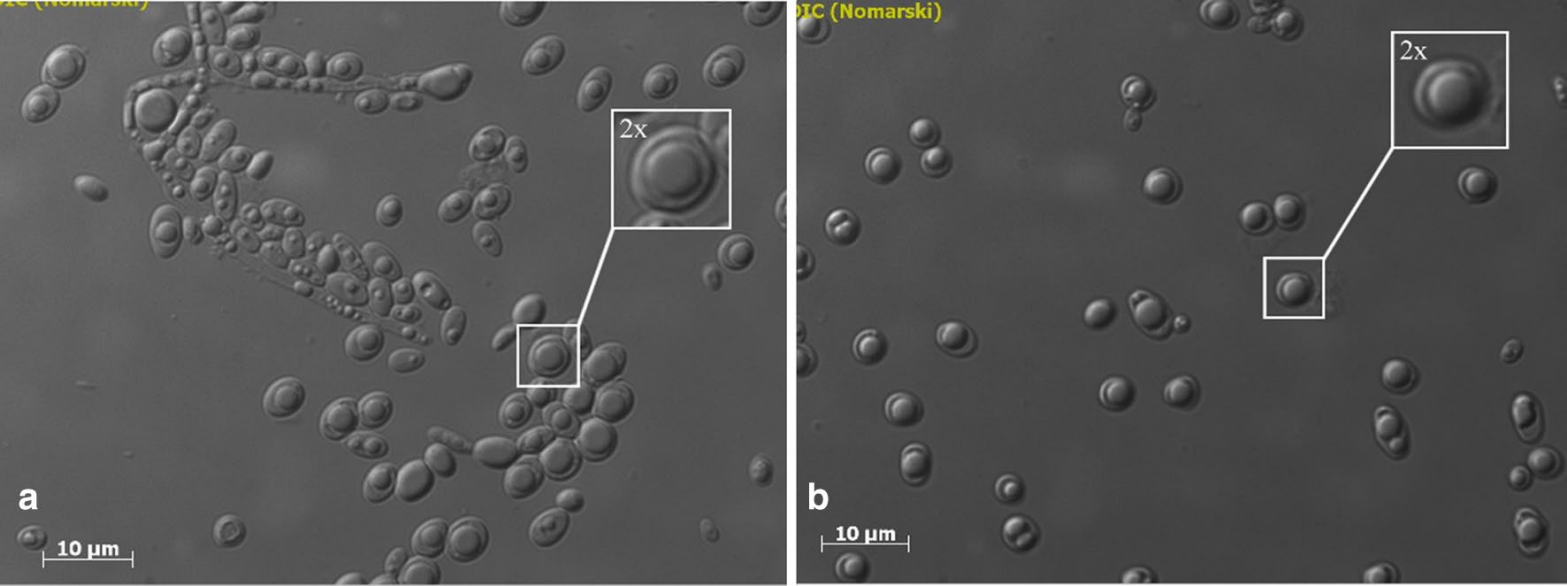
Cell morphology of JMY4086 when the strain was continuously cultured in crude glycerol: **a** at 200 h and **b** at 400 h. The *white squares* show a representative cell that has been enlarged (×2).

The SCFB culturing experiment showed that feed glycerol concentrations of 250 g/L yielded the highest *Q*_*L*_ and *Y*_*L*/*X*_ values. In addition, citric acid and glycerol did not accumulate under those conditions; DO levels exceeded 70% saturation and no mycelia were observed. To assess lipid production in continuous cultures, yeasts were grown in a chemostat for over 400 h using a dilution rate of 0.01 h^−1^. To start off, yeasts were batch cultured for 48 h using molasses as the primary carbon source. Then, chemostat culturing was used; yeasts were kept in a medium with a glycerol concentration of 100 g/L for 100 h. They were then given a medium with a glycerol concentration of 250 g/L (Figure 5). At a steady state, between 200 h and 500 h of culture, the biomass yield was 59.8 g/L. The yeast produced 24.2 g/L of lipids; *Q*_L_ was 0.43 g/L/h; and *Y*_*L*/gly_ and *Y*_*L*/*X*_ were 0.1 and 0.4_,_ respectively (Table 2). Under these conditions, citric acid production was 50 g/L (Figure 5; Table 2). In contrast to the SCFB experiments, citric acid was produced from the start in the chemostat culture. One hypothesis explaining this difference between the two culture methods is that, in the chemostat, DO was limited. During SCFB culture, DO was not a limiting factor until 600 h into the experiment, when citric acid began to be secreted into the culture broth. However, in the chemostat culture, when the glycerol concentration in the feeding medium was increased to 250 g/L, DO dramatically decreased. DO limitations resulted in citric acid secretion, but not in cell filamentation. The cell morphology was constant; indeed, during the whole culturing process, cells remained in yeast form (Figure 6). Fatty acid profiles were similar across the three types of cultures (fed-batch, SCFB, and chemostat; Table 3). The yeast produced mainly C16 and C18 long-chain fatty acids, as do other oleaginous yeasts [25, 32]. In general, differences in fatty acid profiles seem to result not from culture type, but from substrate type. When industrial fats have been used as carbon sources, yeast demonstrates a different total fatty acid composition, which is characterized by high levels of cellular stearic acid [3].

**Table 3:** Fatty acid profiles for *Y. lipolytica* JMY4086 under different culture conditions

Culture method	C 16:0	C (16:1)	C (18:1)	C (18:2)	Other (%)
	Palmitic acid (%)	Palmitoleic acid (%)	Oleic acid (%)	Linoleic acid (%)	
Fed batch	21.4 ± 1.5	9.4 ± 1.9	48.2 ± 0.8	10.3 ± 1.3	10.8 ± 1.2
SCFB	21.0 ± 0.9	8.4 ± 0.6	50.1 ± 1.9	10.0 ± 0.9	9.7 ± 2.0
Chemostat	20 ± 0.5	9.3 ± 1.0	51.0 ± 0.8	10.4 ± 0.7	8.9 ± 0.6

The results of this study represent a good starting point for research seeking to further optimize chemostat culturing conditions. We found that the concentration of dissolved oxygen is one of the most important factors affecting lipid production. Oxygen limitation can be rate limiting in carbon metabolism and results in citric acid secretion. Additionally, optimizing nitrogen levels in the medium is also an important means by which citric acid secretion can be restricted. However, increasing nitrogen concentration can also increase biomass production, which in turn can result in problems with the oxygenation and stirring of the medium. It is therefore important to balance the regulation of available nitrogen with the optimization of the dilution rate to avoid generating overly high biomass concentrations in the bioreactor. All of these parameters should be used to find the right equilibrium between biomass and lipid production, the goal being to maximize total lipid production using chemostat culturing.

## Conclusions

In conclusion, the results obtained in this study clearly show that the continuous culture method is an interesting means of producing lipids. Overall lipid production in the continuous culture experiment was almost 2.3 times higher than that in the fed-batch culture experiment. *Y. lipolytica* JMY4086 produced 24.2 g/L of lipids; the coefficient of lipid yield to glycerol consumption (*Y*_*L*/gly_) was 0.1 g/g and volumetric lipid productivity (*Q*_*L*_) was 0.43 g/L/h. In the fed-batch cultures, lipid concentrations never exceeded 15.5 g/L, which corresponded to a *Y*_*L*/gly_ of 0.083 g/g and a *Q*_L_ of 0.18 g/L/h.

Bioengineered *Y. lipolytica* strain JMY4086 shows promise in the development of industrial biodiesel production processes. Indeed, in this strain, the inhibition of the degradation and remobilization pathways (via the deletion of the six *POX* genes and the *TGL4* gene, respectively) was combined with the boosting of lipid synthesis pathways (via overexpression of *DGA2* and *GPD1*). Additionally, molasses is an excellent substrate for biomass production, because it is cheap and contains several other compounds that are crucial for the fermentation processes. However, its concentration must be controlled because it also contains compounds that inhibit *Y. lipolytica* growth. Moreover, the subsequent addition of glycerol did not delay cell growth. This study provided valuable foundational knowledge that can be used in future studies to further optimize lipid production in fed-batch and continuous cultures in which biomass production takes place in molasses and lipid production takes place in industrial glycerol-based medium.

## Methods

### Strain

The *Y. lipolytica* strain used in this study, JMY4086 [17], was obtained by deleting the *POX1*–*6* genes (*POX1*–*POX6*) that encode acyl-coenzyme A oxidases and the *TGL4* gene, which encodes an intracellular triglyceride lipase. The aim was to block the β-oxidation pathway and inhibit TAG remobilization, respectively. In addition, to push and pull TAG biosynthesis, *YlDGA2* and *YlGPD1*, which encode the major acyl-CoA:diacylglycerol acyltransferase and glycerol-3-phosphate dehydrogenase, respectively, were constitutively overexpressed. Additionally, the *S.cerevisiae* invertase *SUC2* and *Y. lipolytica* hexokinase *HXK1* genes were overexpressed to allow the strain to grow in molasses.

### Medium and culturing conditions

The YPD medium contained Bacto™ Peptone (20 g/L, Difco, Paris, France), yeast extract (10 g/L, Difco, Paris, France), and glucose (20 g/L, Merck, Fontenay-sous-Bois, France). The medium for the batch cultures contained molasses (245 g/L, sucrose content of 600 g/L, Lesaffre, Rangueil, France), NH_4_Cl (4.0 g/L), KH_2_PO_4_ (0.5 g/L), MgCl_2_ (1.0 g/L), and YNB (without amino acids and ammonium sulfate, 1.5 g/L, Difco). For fed-batch cultures, crude glycerol (96% w/v, Novance, Venette, France) was added after 48 h at a feeding rate of 8.8 g/h until a total of 100 g/L of glycerol had been delivered (C:N ratio of 100:1). In the stepwise continuous fed-batch (SCFB) cultures, a C:N ratio of 60:1 was maintained as glycerol concentrations increased (100, 200, 250, 350, and 450 g/L); NH_4_Cl ranged from 4 to 12.5 g/L. Chemostat cultures were grown in either crude glycerol (100 g/L)/NH_4_Cl (2.5 g/L), with a C:N ratio of 25, or glycerol (250 g/L)/NH_4_Cl (6,25 g/l), with a C:N ratio of 40. Toward the beginning of the culturing process (at 100 h), the concentration of glycerol in the feeding medium was 100 g/L; it was subsequently increased to 250 g/L. This approach was used because past observations had suggested that slowly increasing the concentration of the carbon source in the feeding medium results in greater oxygenation of the culture and higher lipid production. Additionally, the fact that the carbon source was present in high concentrations from the beginning of the culturing process resulted in strong cell filamentation and lower final lipid yields (data not shown). For the stepwise continuous fed-batch and chemostat cultures, the dilution rate was 0.01 h^−1^ and the working volume was maintained at 1.5 L. All culturing took place in a 5-L stirred tank reactor (Biostat B-plus, Sartorius, Germany). The temperature was controlled at 28°C and the pH was kept at 3.5 by adding 40% (W/V) NaOH. We used three oxygenation conditions in our experiments: unregulated dissolved oxygen (DO), “Oxy-const”, regulated DO, “Oxy-regul”, and high DO, “Oxy-high” conditions. For the unregulated condition, the airflow rate was 1.5 L/min and the stirring speed was 800 rpm. In the regulated condition, DO was maintained at 50% saturation by a PID controller (the airflow rate ranged between 0 and 3.5 L/min, and stirring speed ranged between 200 and 1,000 rpm). In the oxy-high condition, the airflow rate was 3.5 L/min and the stirring speed was 800 rpm. Bioreactors were inoculated using samples with an initial OD_600 nm_ of 0.15 (low-density inoculum) or of 0.8 (high-density inoculum). Precultures were grown in YPD medium. The bioreactor containing a given medium (prepared with tap water) was sterilized in an autoclave at 121°C for 20 min. We conducted two biological replicates of all fed-batch cultures, for which means and standard deviations were calculated. A single replicate was performed for the SCFB and the chemostat culture.

### Analytical methods

#### Quantifying dry biomass

Ten milliliters of culture broth was centrifuged for 5 min at 13,000 rpm. The cell pellet was washed with distilled water and filtered on membranes with a pore size of 0.45 μm. The biomass yield was determined gravimetrically after samples were dried at 105°C. It was expressed in grams of cell dry weight per liter (gCDW/L).

#### Measuring sugar and citric acid concentrations

The concentrations of glycerol (GLY), sucrose (SUC), glucose (GLU), fructose (FRU), and citric acid (CA) were measured in the culture supernatants by HPLC (Dionex-Thermo Fisher Scientific, UK) using an Aminex HPX-87H column (Bio-Rad, Hercules, CA, USA) coupled with a refractive index (RI) detector (Shodex, Ogimachi, Japan). The column was eluted with 0.1 N sulfuric acid at 65°C at a flow rate of 0.6 ml min^−1^.

#### Fluorescence microscopy

Images were obtained using a Zeiss Axio Imager M2 microscope (Zeiss, Le Pecq, France) with a 100× objective lens and Zeiss filter sets 45 and 46 for fluorescence microscopy. Axiovision 4.8 software (Zeiss, LePecq, France) was used for image acquisition. To make the lipid bodies visible, BodiPy^®^ Lipid Probe (2.5 mg/mL in ethanol; Invitrogen) was added to the cell suspension (OD_600_ = 5) and the samples were incubated for 10 min at room temperature.

#### Quantifying lipid levels

The fatty acids (FAs) in 15-mg aliquots of freeze-dried cells were converted into methyl esters using the method described in Browse et al. [34, 30]. FA methyl esters were analyzed by gas chromatography (GC) on a Varian 3900 equipped with a flame ionization detector and a Varian Factor Four vf-23 ms column, for which the bleed specification at 260°C was 3 pA (30 m, 0.25 mm, 0.25 μm). FAs were identified by comparing their GC patterns to those of commercial FA methyl ester standards (FAME32; Supelco) and quantified using the internal standard method, which involved the addition of 50 mg of commercial C17:0 (Sigma). Total lipid extractions were obtained from 100-mg samples (expressed in terms of CDW, as per Folch et al. [16]). Briefly, yeast cells were spun down, washed with water, freeze dried, and then resuspended in a 2:1 chloroform/methanol solution and vortexed with glass beads for 20 min. The organic phase was collected and washed with 0.4 mL of 0.9% NaCl solution before being dried at 60°C overnight and weighed to quantify lipid production.

#### Calculations

Volumetric lipid productivity (*Q*_*L*_) was defined using Eqs. (1–3):

1 $$Q_{\text{L}} = \frac{{{\text{Lipid}}_{\text{acc}} + {\text{Lipid}}_{\text{out}} }}{V \Delta t},$$

2 $${\text{Lipid}}_{\text{acc}} = \left[ {\text{Lipid}} \right] . X,$$

3 $${\text{Lipid}}_{\text{out}} = \frac{{\Delta \left( {{\text{Lipid}}_{\text{acc}} } \right)}}{F \Delta t}.$$

The coefficient of lipid yield to glycerol consumption (*Y*_*L*/gly_) was defined using Eqs. (4–7):

4 $$Y_{{L/{\text{gly}}}} = \frac{{{\text{Lipid}}_{\text{acc}} + {\text{Lipid}}_{\text{out}} }}{{{\text{Gly}}_{\text{in}} - ({\text{Gly}}_{\text{acc}} + {\text{Gly}}_{\text{out}} )}},$$

5 $${\text{Gly}}_{\text{in}} = \left[ {\text{Gly}} \right] . F. \Delta t,$$

6 $${\text{Gly}}_{\text{Acc}} = \Delta \left[ {{\text{Gly}}_{\text{med}} } \right] . V,$$

7 $${\text{Gly}}_{\text{out}} = \frac{{\Delta \left( {{\text{Gly}}_{\text{acc}} } \right)}}{F \Delta t}.$$

The coefficient of lipid yield to biomass yield (*Y*_*L*/*X*_) was defined using Eqs. (8–10):

8 $$Y_{L/X} = \frac{{{\text{Lipid}}_{\text{acc}} + {\text{Lipid}}_{\text{out}} }}{{X_{\text{in}} + X_{\text{out}} }},$$

9 $$X_{\text{in}} = \Delta \left[ X \right] . V,$$

10 $$X_{\text{out}} = \frac{{\Delta \left( {X_{\text{in}} } \right)}}{F \Delta t}.$$

In the above equations, Lipid_acc_ is the lipid accumulated in the cells in the bioreactor (g); Lipid_out_ the lipid accumulated in the cells drawn off from the bioreactor (g); Δ(lipid_acc_) the difference in lipid_acc_ for time period Δ*t*; Gly_in_ the glycerol fed to the bioreactor (g); Gly_acc_ the glycerol accumulated in the bioreactor (g); Gly_out_ the glycerol drawn off from the bioreactor (g); Δ(Gly_acc_) the difference in Gly_acc_ for the time period Δ*t*; *V* the volume of the culture (L); Δ*t* the duration between two measurements (h); *X* the biomass yield (gCDW/L); [Gly] the glycerol concentration in the feeding medium (g/L); [Gly_med_] the glycerol concentration in the bioreactor (g/L); [Lipid] the lipid concentration (g/CDW); [*X*_in_] the cell concentration in the bioreactor (gCDW/L); [*X*_out_] the cell concentration in the culture broth drawn off from the bioreactor (gCDW/L); *F* the flow rate of the feeding medium (L/h).

## Abbreviations

*Y*_*L*/gly_: coefficient of lipid yield to glycerol consumption
*Y*_*L*/*X*_: coefficient of lipid yield to biomass yield
*Q*_*L*_: volumetric lipid productivity
*X*: biomass yield
CDW: cell dry weight
SCFB: stepwise continuous fed batch
GLY: glycerol
CA: citric acid
*L*: intracellular lipid
*D*: dilution rate
DO: dissolved oxygen
rpm: rotations per minute

## References

[1] Tai M, Stephanopoulos G (2013). Engineering the push and pull of lipid biosynthesis in oleaginous yeast *Yarrowia lipolytica* for biofuel production. Metab Eng.

[2] Hill J, Nelson E, Tilman D, Polasky S, Tiffany D (2006). Environmental, economic, and energetic costs and benefits of biodiesel and ethanol biofuels. Proc Natl Acad Sci USA.

[3] Papanikolaou S, Chevalot I, Komaitis M, Aggelis G, Marc I (2001). Kinetic profile of the cellular lipid composition in an oleaginous *Yarrowia lipolytica* capable of producing a cocoabutter substitute from industrial fats. Antonie Van Leeuwenhoek.

[4] Papanikolaou S, Chevalot I, Komaitis M, Marc I, Aggelis G (2002). Single cell oil production by *Yarrowia lipolytica* growing on an industrial derivative of animal fat in batch cultures. Appl Microbiol Biotechnol.

[5] Meher LC, Sagar DV, Naik SN (2006). Technical aspects of biodiesel production by transesterification—a review. Renew Sust Energy Rev.

[6] Beopoulos A, Cescut J, Haddouche R, Uribelarrea JL, Molina-Jouve C, Nicaud JM (2009). *Yarrowia lipolytica* as a model for bio-oil production. Prog Lipid Res.

[7] Beopoulos A, Nicaud JM (2012). Yeast: a new oil producer. Ol Corps Gras Lipides OCL.

[8] Thevenieau F, Nicaud J-M (2013). Microorganisms as sources of oils. Ol Corps Gras Lipides OCL.

[9] Mliĉková K, Luo Y, Andrea S, Peĉ P, Chardot T, Nicaud JM (2004). Acyl-CoA oxidase, a key step for lipid accumulation in the yeast *Yarrowia lipolytica*. J Mol Catal B Enzym.

[10] Beopoulos A, Mrozova Z, Thevenieau F, Dall MT, Hapala I, Papanikolaou S (2008). Control of lipid accumulation in the yeast *Yarrowia lipolytica*. Appl Environ Microbiol.

[11] Blazeck J, Hill A, Liu L, Knight R, Miller J, Pan A (2014). Harnessing *Yarrowia lipolytica* lipogenesis to create a platform for lipid and biofuel production. Nat Commun.

[12] Li Y, Zhao ZK, Bai F (2007). High-density cultivation of oleaginous yeast *Rhodosporidium toruloides* Y4 in fed-batch culture. Enzyme Microb Tech.

[13] Zhao X, Kong X, Hua Y, Feng B, Zhao ZK (2008). Medium optimization for lipid production through co-fermentation of glucose and xylose by the oleaginous yeast *Lipomyces starkeyi*. Eur J Lipid Sci Technol.

[14] Meesters PAEP, Huijberts GNM, Eggink G (1996). High-cell-density cultivation of the lipid accumulating yeast *Cryptococcus curvatus* using glycerol as a carbon source. App Microbiol Biotechnol.

[15] Papanikolaou S, Aggelis G (2002). Lipid production by *Yarrowia lipolytica* growing on industrial glycerol in a single-stage continuous culture. Bioresour Technol.

[16] Barth G, Gaillardin C, Wolf K (1996). *Yarrowia lipolytica*. Nonconventional yeasts in biotechnology.

[17] Xie D, Jackson EN (2015). Zhu Q Sustainable source of omega-3 eicosapentaenoic acid from metabolically engineered *Yarrowia lipolytica*: from fundamental research to commercial production. Appl Microbiol Biotechnol.

[18] Folch J, Lees M, Sloane-Stanley GH (1957). A simple method for the isolation and purification of total lipids from animal tissues. J Biol Chem.

[19] Lazar Z, Dulermo T, Neuvéglise C, Crutz-LeCoq A-M, Nicaud J-M (2014). Hexokinase—a limiting factor in lipid production from fructose in *Yarrowia lipolytica*. Metab Eng.

[20] Joshi S, Bharucha C, Jha S, Yadav S, Nerurkar A, Desa AJ (2008). Biosurfactant production using molasses and whey under thermophilic conditions. Bioresour Technol.

[21] Makkar RS, Swaranjit S, Cameotra SS (1997). Utilization of molasses for biosurfactant production by two *Bacillus* strains at thermophilic conditions. J Am Oil Chem Soc.

[22] Babel W, Hofmann KH (1981). The conversion of triosephosphate via methylglyoxal, a bypass to the glycolytic sequence in methylotrophic yeasts?. FEMS Microbiol Lett.

[23] Ermakova IT, Morgunov IG (1987). Pathways of glycerol metabolism in *Yarrowia (Candida) lipolytica* yeasts. Mikrobiology.

[24] May JW, Sloan J (1981). Glycerol utilization by *Schizosaccharomyces pombe*: dehydrogenation as the initial step. J Gen Microbiol.

[25] Makri A, Fakas S, Aggelis G (2010). Metabolic activities of biotechnological interest in *Yarrowia lipolytica* grown on glycerol in repeated batch cultures. Bioresour Technol.

[26] Bellou S, Makri A, Triantaphyllidou I-E, Papanikolaou S, Aggelis G (2014). Morphological and metabolic shifts of *Yarrowia lipolytica* induced by alteration of the dissolved oxygen concentration in the growth environment. Microbiology.

[27] Ykema A, Verbree EC, Verseveld HW, Smit H (1986). Mathematical modeling of lipid production by oleaginous yeast in continuous cultures. Antoinie Van Leeuwenhoek.

[28] Brown BD, Hsu KH, Hammond EG, Glatz BA (1989). A relationship between growth and lipid accumulation in *Candida cyrvata* D. J Ferment Bioeng.

[29] Ratledge C, Kamel BS, Kakuda Y (1994). Yeast moulds algae and bacteria as sources of lipids. Technological advances in improved and alternative sources of lipids.

[30] Evans CT, Ratledge C (1983). A comparison of the oleaginous yeast *Candia curvata* grown on different carbon sources in continuous and batch culture. Lipids.

[31] Rywińska A, Juszczyk P, Wojtatowicz M, Rymowicz W (2011). Chemostat study of citric acid production from glycerol by *Yarrowia lipolytica*. J Biotechnol.

[32] Davies RJ, Ratledge C, Kyle DJ (1992). Scale up of yeast oil technology. Industrial application of single cell oil.

[33] Meesters PAEP, van de Wal H, Weusthuis R, Eggink G (1996). Cultivation of the oleaginous yeast *Carptococcus curvatus* in a new reactor with improved mixing and mass transfer characteristics (surer^®^). Biotechnol Tech.

[34] Browse J, Mc Court PJ, Somerville CR (1986). Fatty acid composition of leaf lipids determined after combined digestion and fatty acid methyl ester formation from fresh tissue. Anal Biochem.

